# Single-cell RNA sequencing of the post–spinal cord injury dorsal root ganglia in cynomolgus monkeys: Elucidation of the cellular immune microenvironment of the central nervous system

**DOI:** 10.4103/NRR.NRR-D-24-00974

**Published:** 2025-03-25

**Authors:** Yiming Ren, Bo Li, Bo Yang, Baoyou Fan, Shenghui Huang, Guidong Shi, Liang Liu, Zhijian Wei, Shiqing Feng

**Affiliations:** 1 Department of Orthopedics, International Science and Technology Cooperation Base of Spinal Cord Injury, Tianjin Key Laboratory of Spine and Spinal Cord Injury, Tianjin Medical University General Hospital, Tianjin, China; 2 Department of Joint and Sport Medicine, Tianjin Union Medical Center, The First Affiliated Hospital of Nankai University, Tianjin, China; 3 Department of Orthopedics, Beijing Luhe Hospital, Capital Medical University, Beijing, China; 4 Department of Othopedics, Qilu Hospital of Shandong University, Jinan, Shandong Province, China

**Keywords:** cellular communication, cellular microenvironment, differentially expressed genes, dorsal root ganglia, immune cells, macrophage, microglia, neurons, single-cell sequence, spinal cord injury

## Abstract

Few studies have investigated alterations in the immune cell microenvironment of the dorsal root ganglia following spinal cord injury and whether these modifications facilitate axonal regeneration. In this study, we used a single-cell RNA sequencing dataset to create a comprehensive profile of the diverse cell types in the dorsal root ganglia and spinal cord of a mid-thoracic contusion injury model in cynomolgus monkeys. Cell communication analysis indicated that specific signaling events among various dorsal root ganglia cell types occur in response to spinal cord injury. Single-cell analysis using dimensionality reduction clustering identified distinct molecular signatures for nine cell types, including macrophage subpopulations, and differential gene expression profiles between dorsal root ganglia cells and spinal cord cells following spinal cord injury. The macrophage subpopulations were categorized into 11 clusters (MC0–MC10) based on differentially expressed genes, with the top 10 genes being *ABCA6*, *RBMS3*, *EBF1*, *LAMA4*, *ANTXR2*, *LAMA2*, *SOX5*, *FOXP2*, *GHR*, and *APOD*. MC0, MC1, and MC2 constituted the predominant macrophage populations. MC4, MC6, and MC9 were nearly absent in the spinal cord, but exhibited significant increases in the dorsal root ganglia post–spinal cord injury. Notably, these subpopulations possess a strong capacity for regulating axonal regeneration. The developmental progression of dorsal root ganglia macrophages after spinal cord injury was elucidated using cell trajectory and pseudo-time analyses. Genes such as *EBF1* (MC6 and MC9 marker), *RBMS3* (MC6 and MC9 marker), and *ABCA6* (MC6 marker) showed high expression levels in the critical pathways of macrophage function. Through ligand–receptor pair analysis, we determined that the effects of macrophages on microglia are predominantly mediated through interaction pairs (e.g., SPP1-CD44, LAMC1-CD44, and FN1-CD44), potentially facilitating specific cellular communications within the immune microenvironment. The single-cell RNA sequencing dataset used in this study represents the first comprehensive transcriptional analysis of the dorsal root ganglia after spinal cord injury in cynomolgus monkeys, encompassing nearly all cell types within the dorsal root ganglia region. Using this dataset, we evaluated diverse subtypes of macrophages in the post- spinal cord injury dorsal root ganglia area and examined the signaling pathways that facilitate interactions among immune response-related macrophages in the dorsal root ganglia. Findings from this study provide a theoretical basis for understanding how the immune microenvironment influences the regenerative capacity of dorsal root ganglia neurons after spinal cord injury and offer novel insights into the complex processes underlying the pathobiology of spinal cord injury.

## Graphical abstract

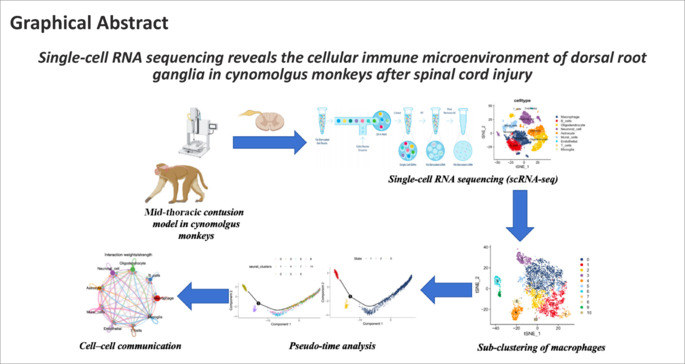

## Introduction

Spinal cord injury (SCI) frequently results in reduced or lost sensation, mobility, and reflexes below the injury site, autonomic nervous system dysfunction, urinary and fecal incontinence, and profound disability (Fan et al., 2018; Huang et al., 2020; Guo et al., 2021). Newly identified characteristics of SCI include a high incidence (3.0–3.5/100,000 in the USA), a substantial rate of disability (67% experiencing total paralysis), considerable costs (50,000–70,000 US dollars per patient per year in the USA), and a low mortality rate (< 5%) (Gatti et al., 2020; Mesbah et al., 2021; Kao et al., 2022; Wecht et al., 2023; Zheng et al., 2023; Hiremath et al., 2024; Lee et al., 2024).

While central nervous system (CNS) injuries, including SCI, exhibit extremely limited regeneration, the peripheral nervous system (PNS) and embryonic nervous system axons display notable regenerative ability. One primary factor contributing to the varying regenerative abilities of axons is the distinctly different microenvironments of the PNS and CNS. Following CNS injury, various axonal growth inhibitory molecules are present in the microenvironment (Filbin, 2003; Hao et al., 2024). Furthermore, immune cells and/or glial cells influence the regeneration of damaged axons in the injured CNS (O’Shea et al., 2017). The activated inflammatory response to an SCI functions to protect against pathogens and facilitate tissue recovery to maintain cellular homeostasis. However, excessive inflammation may result in pathological alterations to the immune system, leading to cellular dysregulation and chronic diseases (Bennett et al., 2018). The dual aspect of the inflammatory response is also reflected in the diverse roles of immune cells, including astrocytes, microglia, and macrophages. Microglia contribute to maintaining local homeostasis and are rapidly mobilized alongside macrophages to initiate innate immune responses (Kettenmann et al., 2011; Yang et al., 2025). By contrast, fewer axonal inhibitory factors are produced following PNS injury, with environmental factors significantly influencing the success or failure of regeneration. The regenerative potential of central neurons may be realized only when the CNS neuroglial environment is altered to resemble that of the PNS (Aguayo et al., 1981; David and Aguayo, 1981).

Single-cell RNA sequencing (scRNA-seq) facilitates the analysis of biological functions by examining the transcriptomes of individual cells (Kolodziejczyk et al., 2015; Wagner et al., 2016), enabling impartial analysis of cell population characteristics within damaged tissues. Milich et al. (2021) used scRNA-seq to assess virtually all cell types that comprise the mouse SCI site, providing novel mechanistic insights into the pathobiology of SCI and other traumatic disorders of the CNS. However, scRNA-seq of the CNS alone cannot elucidate the differences in the neural microenvironment between the CNS and PNS. Dorsal root ganglia (DRG) comprise immune cells and/or glial cells and the somata of primary sensory neurons. Following SCI, specific groups of DRG neurons exhibit heightened sensitivity and undergo the growth of new axon branches both in the PNS and CNS (Chariker et al., 2019). However, few studies have focused on the post-SCI changes in the microenvironment of immune cells in the DRG, and whether they contribute to axonal regeneration in DRG neurons. Furthermore, the impact of microenvironment alterations on local cellular composition, the course of cell maturation, and cell-to-cell communication at the level of individual cells remains yet to be fully explored. Avraham et al. (2021) demonstrated that the mouse DRG microenvironment responds differently to central and peripheral axon injuries, indicating that the manipulation of non-neuronal cells could offer new avenues for promoting functional recovery after CNS injuries. However, the non-neuronal immune microenvironment of mice is not entirely homologous to that of humans.

The majority of pathological findings in animal models of SCI have originated from pigs, dogs, and rodents, including mice. These findings have significantly advanced fundamental research focused on repairing and safeguarding neurological function, and promoting regeneration after SCI. Although mice are frequently used for basic research owing to their genetic homology with humans (Crowe et al., 1997; Liu et al., 1997; Rossignol et al., 2007), pigs, dogs, and humans possess similar spinal cord tissues, facilitating modeling and evaluation. Zebrafish, whose axons can regenerate after SCI, are often employed as animal models to study SCI, but their neural microenvironment differs from that of humans (Babin et al., 2014). The transition from basic research to clinical applications sometimes requires large animal models, such as non-human primates, to validate the most effective strategies before progressing to clinical trials. This necessity arises from notable variations in neuroanatomical, pathological, and functional characteristics, including disparities in the source, direction, endpoint, and dimensions of key spinal pathways between rodents and humans (Courtine et al., 2007). Furthermore, cellular reactions to SCI, including those of astrocytes, also differ between rodents (Wang et al., 2009) and humans (Puckett et al., 1997). Given these disparities, it may be challenging to directly apply research findings from rodents to clinical settings. With the remarkable similarity in anatomy and function between non-human primates and humans, elucidation of the post-SCI pathology of non-human primates, both in the acute and chronic phases, could significantly enhance preclinical exploration of therapeutic approaches. Regrettably, apart from initial reports of axonal pathology observed at the light and electron microscopic levels in monkey models, relatively few investigations have been conducted to assess systemic pathological alterations in the monkey post SCI (Bresnahan et al., 1976; Bresnahan, 1978).

To address this research question, we performed scRNA-seq to generate a comprehensive dataset of diverse cell types present in DRG and the spinal cord following SCI using a cynomolgus monkey mid-thoracic contusion model. As the first scRNA-seq assessment of all DRG cells following SCI in cynomolgus monkeys, our transcriptomic dataset will support further investigation into the functional recovery role of the post-SCI DRG microenvironment.

## Methods

### Animals and surgical procedure

All experiments were approved by the Institutional Animal Care and Use Committee of the Research Center for Drug Safety Evaluation of Hainan Province (Ethical Code 20220915-NHP [02]) on September 12, 2022. All experiments were designed and reported in accordance with the Animal Research: Reporting of *In Vivo* Experiments (ARRIVE) guidelines (Percie du Sert et al., 2020), and The Weatherall report on the use of non-human primates in research.

Two adult female cynomolgus monkeys (body weights: 4.9 and 5.1 kg; age: 5 years) were used in this study: one served as the untreated control, while the other was used to establish the SCI model. Both animals were provided by and housed at the Institutional Animal Care and Use Committee of the Research Center for Drug Safety Evaluation of Hainan Province (License No. SYXK (Qiong) 2017-0013), where the surgical procedures were also conducted. Anesthesia was initiated with intravenous (i.v.) injection of propofol (2.5 mg/kg; AstraZeneca PLC, London, UK) and maintained with i.v. propofol (5 mg/kg/h) in combination with remifentanil (0.3 mg/kg/h; Renfu Pharmaceutical Co., Ltd., Yichang, China). Oxygen was supplied at a rate of 1.5 L/min through an endotracheal tube. The animals received a prophylactic intramuscular (i.m.) injection of the antibiotic ceftriaxone sodium (50 mg/kg/24 hours; Rocephin®, Roche Pharmaceutical Co., Ltd., Shanghai, China) within 72 hours postoperatively.

**Figure 1:**
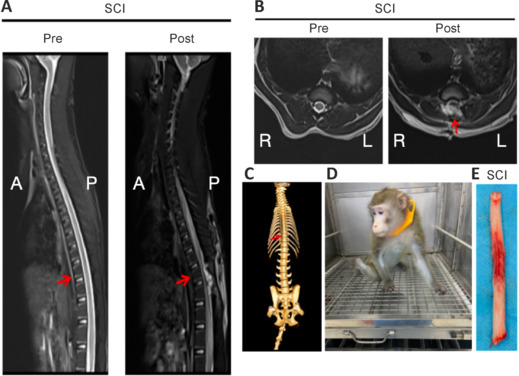
Establishment of the SCI model in a cynomolgus monkey. (A, B) Magnetic resonance imaging showing the status of the spinal cord before and after animal modeling in sagittal (A) and axial (B) T2-weighted images. The pre-SCI T2-weighted image shows low signal intensity in the spinal cord, while the post-SCI image shows high signal intensity due to spinal cord contusion and edema. The red arrow indicates the SCI. (C) Three-dimensional CT imaging illustrating the modeling position. The red arrow indicates the location where the spinous process and vertebral plate are surgically removed. (D) The SCI model monkey that was placed back in its cage for dynamic monitoring of vital signs. (E) The segment of spinal cord tissue that was dissected from the center of the SCI site. A: Anterior; CT: computed tomography; L: left; P: posterior; R: right; SCI: spinal cord injury.

To establish the SCI model, the spinal processes and vertebral laminae between T7 and T9 were surgically excised, exposing the T8 spinal cord near the T8 vertebra of the monkey. Following adequate exposure of the spinal cord, a contusion injury was induced by dropping a 50-g weight from a height of 50.0 mm using the SS-II spinal cord contusion impactor (Institutional Animal Care and Use Committee of Research Center, Hainan, China). The monkey’s vital signs were monitored in accordance with standard post-anesthesia care protocols typically employed for humans. Neurological assessments were conducted following initial extubation and recovery from anesthesia. Upon regaining consciousness, the animals were returned to their cages, with a mattress placed at the bottom to minimize the risk of pressure sores. Imaging was performed to evaluate and confirm the successful modeling (**Figure 1**). The monkeys were administered i.v. doses of antibiotics and glucose (both from Beijing Biolab Technology Co. Ltd., Beijing, China) for a minimum of 3 days. The wounds were treated with 3 mL of 2% povidone-iodine (Beijing Biolab Technology Co. Ltd.) daily. The animals were examined twice a day to evaluate skin health and monitor any signs of muscle atrophy, a potential consequence of limb denervation. To prevent urine retention, manual bladder expression and gentle abdominal massages were performed. At 3 days post injury, the animals were humanely euthanized for examination using heavy sedation with ketamine hydrochloride (40 mg/kg; Fangming Pharmaceutical Co., Ltd., Hezhe, Shandong, China) administered via i.m. injection. Once it was confirmed that they had no reflex response to tactile stimuli, the monkeys underwent transcardiac perfusion with 3000 mL of saline solution, followed by a 3000-mL infusion of a 4% paraformaldehyde solution in normal saline.

### Single-nucleus/cell suspension preparation

A 5-mm segment of spinal cord and a 2-mm segment of DRG were excised from the central area at the T8 level, rinsed with Hank’s balanced salt solution, and then soaked with collagenase for 20 minutes at 37°C. The specimens were then physically separated into individual cells via gentle crushing in Neurobasal medium supplemented with GlutaMAX, penicillin-streptomycin, and B-27 (Gibco, New York, NY, USA). For RNA isolation, the spinal cord and DRG tissues were finely chopped and subsequently treated with 2 mL collagenase (1 mg/mL in phosphate-buffered saline [PBS]) for 30 minutes at 37°C, followed by centrifugation at 300 × *g* for 5 minutes at 4°C. The resultant cell pellet was resuspended in 2 mL 0.25% trypsin-ethylene diamine tetraacetic acid solution and incubated at 37°C for 5 minutes. Trypsin digestion was stopped by adding 10% fetal bovine. The mixture was centrifuged, and the obtained pellet was dispersed in PBS containing 0.5% bovine serum albumin, then passed through a 40-µm cell strainer twice to eliminate large cell aggregates. To minimize the presence of blood cells, 10 mL erythrocyte lysis buffer was added to the cell suspension for 10 minutes, and centrifuged. The cells were collected, resuspended in PBS, and the single-cell suspension was used for cell counting.

### scRNA-seq analysis

For the 10X Genomics data, the Cell Ranger Single-Cell toolkit (v3.0.0; 10X Genomics, Majorbio Co., Ltd., Shanghai, China) was employed to align the reads and generate the gene–cell unique molecular identifier (UMI) matrix for each sample (https://support.10xgenomics.com/single-cell-gene-expression/software/downloads/latest). The reads were aligned to the corresponding reference genome of Macaca fascicularis (https://www.ncbi.nlm.nih.gov/Taxonomy/Browser/wwwtax.cgi; Schoch et al., 2020). Multiple samples were combined using the Cell Ranger aggr function. The resulting raw_feature_bc_matrix was loaded, and the Seurat R package (v4.0.1; https://cran.r-project.org/web/packages/Seurat/index.html) was subsequently used for downstream analyses. Further quality control was sequentially conducted on cells using three metrics: total UMI count, number of detected genes, and mitochondrial gene percentage per cell. Specifically, cells with > 200 detected genes and mitochondrial gene expression elevated by > 5% were excluded from the analysis. To further eliminate potential doublets, cells with > 2500 detected genes were also removed. After quality control, the data were normalized and scaled using the SCTransform function of Seurat, and the ribosomal gene percentage was regressed. To address batch effects across different individuals, anchors between samples were identified and applied in the IntegrateData function. Data were visualized through dimensionality reduction using uniform manifold approximation and projection (UMAP) or t-distributed stochastic neighbor embedding (t-SNE).

### Cell annotation

The marker genes for each cluster were identified using the FindAllMarkers function from Seurat. Statistical significance of the data was assessed through the model-based analysis of a single-cell transcriptomics algorithm. Genes were considered signature genes if they met the following criteria: 1) an adjusted *P* value < 0.01 after the correction for false discovery rate using all features in the dataset; 2) a log fold-change in average expression > 0.25; and 3) and a percentage of cells expressing the feature in the first group (pct.1) value > 0.25. Functional enrichment analysis was subsequently conducted on these genes to identify cell type-specific pathways. The primary cell types were identified using the SingleR package (https://bioconductor.org/packages/devel/bioc/html/SingleR.html; Aran et al., 2019). Next, manual verification was performed to ensure that the identified markers were expressed specifically in the corresponding clusters using the criteria of pct.1 > 0.6 and pct.2 < 0.4. The same methodology was applied to subclustering for variable gene detection, dimensionality reduction, and cell integration.

### Differential gene analysis and functional annotation

Differentially expressed genes (DEGs) were identified using the FindAllMarkers function in Seurat and visualized in heatmaps generated by the pheatmap package. Model-based analysis of the single-cell transcriptomics algorithm was employed to assess statistical significance. DEGs were defined by an absolute log fold-change in average expression > 0.25 and an adjusted *P* value < 0.01. Functional enrichment analysis on these DEGs was conducted using the Kyoto Encyclopedia of Genes and Genomes (KEGG) and Gene Ontology (GO) databases for annotation. GO enrichment analysis was performed using the R clusterProfiler (v3.14.3) package (https://bioconductor.org/packages/release/bioc/html/clusterProfiler.html; Yu, 2024), while pathway enrichment analysis was conducted with the hypergeometric distribution through the R phyper function, applying a significance threshold of corrected *P* values < 0.05.

### Pseudo-time analysis

The Monocle R package (version 2.18.0; https://www.bioconductor.org/packages/3.15/bioc/src/contrib/Archive/monocle/) and the reverse-graph embedding machine learning algorithm were employed to conduct cell trajectory and pseudo-time analyses. Initially, genes indicative of various stages of differentiation were selected for the ordering process. These genes were subsequently arranged using the orderCells function following principal component analysis. Each cell in the high-dimensional space was represented as a point reflecting the expression levels of the ordered genes. Subsequently, a differentiation tree based on the selected data was generated using Monocle 2, and the algorithm directed each cell to the nearest vertex within the tree, adjusting its position to correspond to the cell’s characteristics. This iterative process was used to construct a new spanning tree, continuing until all cells converged. Throughout this process, gene ordering and dimensionality were controlled. Upon tree establishment, Monocle identified a tip as the “root.” The geodesic distance from each cell to the root was computed and branches were automatically allocated. For pseudo-time analysis, differential gene testing was conducted on the basis of the pre-determined cell clusters in the Seurat object, using a significance *P* value < 0.01.

### Cell–cell communication analysis

Interactions among different cell types were investigated using a ligand–receptor analysis. Cellular communication is dependent on interactions between ligands and receptors on the cell surface, as well as with cytokines secreted by the cells. The CellChat package is a comprehensive database that includes data on ligands, receptors, cofactors, and polymers. We employed CellChat (v0.5.5; https://github.com/sqjin/CellChat; Jin et al., 2024) to analyze cell–cell communication. Specifically, the CellChat DB.mouse database, containing ligand–receptor pairs, was employed as a reference list. The clustered cell data from the previous analysis using Seurat were used to prepare the CellChat object. The identifyOverExpressedGenes function was employed to detect highly expressed genes, from which the corresponding pathways were identified. Significant differences in gene expression were evaluated and the probability of ligand–receptor interactions between cells was estimated using the computeCommunprob function. Cells with a communication probability < 20% were excluded using a predefined cutoff parameter. Finally, the outcomes were compared with the imported ligand–receptor database, and alluvial and circle plots were generated for visualization.

### Statistical analyses

Statistical tests were conducted using GraphPad Prism (v10.0.0 for Windows, GraphPad Software, Boston, MA, USA; www.graphpad.com). In all figures, data are represented as means ± standard error of the mean (SEM). Statistical evaluations involved one- or two-way analysis of variance, with a *post hoc* Tukey’s test. A *P* value < 0.05 was considered statistically significant.

## Results

### Single-cell analysis of cell types in the post–spinal cord injury dorsal root ganglia

**Figure 2:**
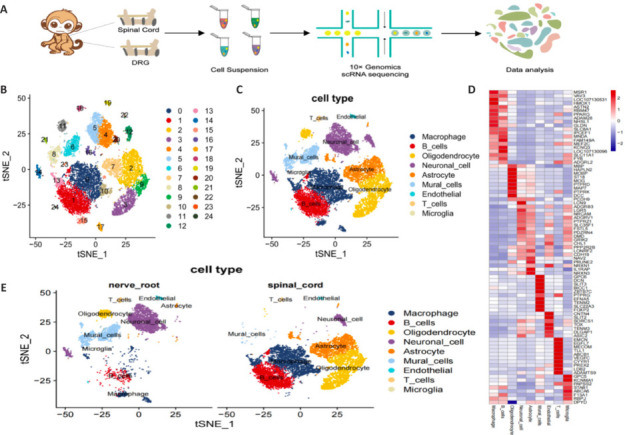
Single-cell dataset with reduced dimension clustering and cell type identification in DRG cells from a cynomolgus monkey with SCI. (A) Schematic representation of the experimental workflow. (B) Cluster analysis of cell groups, and t-SNE distribution showing cluster analysis groupings. Each point represents a cell, and cells that are close in distance are considered to be of the same type. Different groups of cells are distinguished by different colors and numbers. (C) t-SNE plot of all cells collected from the DRG following SCI. Cells are colored and annotated by cell type. (D) Heatmap of distinct genes with high and low expression enriched in each cell type. (E) t-SNE distribution of different cell types in the DRG and spinal cord following SCI. DRG: Dorsal root ganglia; SCI: spinal cord injury; t-SNE: t-distributed stochastic neighbor embedding.

The procedure for SCI and sample collection is illustrated in **Figure 2A**. The scRNA-seq of cells in the DRG and spinal cord was conducted on day 3 following SCI to analyze differences in gene expression. After applying quality control filters, approximately 8918 cells were obtained. After logarithmic normalization, the 2000 most highly variable genes were selected for principal component analysis to reduce dimensionality. The data were normalized, and the first 30 principal components were employed to group cells exhibiting similar gene expression patterns. Several cell clusters were identified and then merged into 25 primary cell groups using t-SNE dimensionality reduction. The cell clusters derived from the post-SCI DRG were visualized on t-SNE plots (**Figure 2B**). The identified cell types were T cells, B cells, neurons, macrophages, microglia, astrocytes, endothelial cells, oligodendrocytes, and mural cells (**Figure 2C**). The DEGs with the highest values for each cell type, determined through the CellMarker database, are shown in **Figure 2D**. The proportions of T cells, neurons, endothelial cells, and mural cells were significantly increased in the DRG compared with those in the spinal cord post SCI. Conversely, the numbers of some immune-related cell types, including B cells, macrophages, microglia, oligodendrocytes, and astrocytes, were decreased in the DRG post SCI (**Figure 2E**).

### Identification of macrophage subtypes and key factors through differentially expressed genes

**Figure 3:**
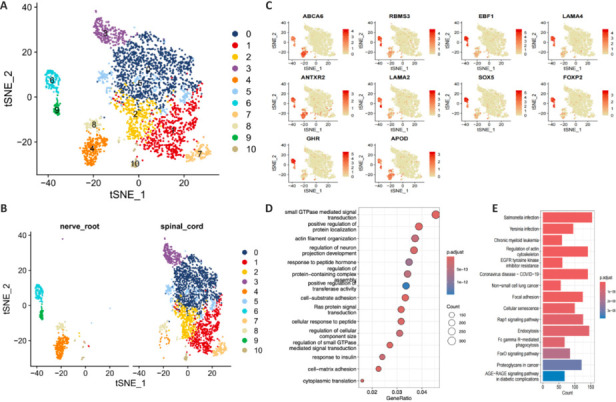
Macrophage subgroup and functional analyses of DRG cells following SCI in a cynomolgus monkey. (A, B) t-SNE plots showing the clusters of macrophage cell subgroups (A) and the distribution of different macrophage subtypes in the DRG and spinal cord (B) following SCI. (C) The 10 DEGs with the highest expression levels in the macrophage subsets. (D) Gene Ontology analysis at the biological process, molecular function, and cellular component levels. (E) Kyoto Encyclopedia of Genes and Genomes enrichment analysis. DEGs: Differentially expressed genes; DRG: dorsal root ganglia; SCI: spinal cord injury; t-SNE: t-distributed stochastic neighbor embedding.

Macrophages play a vital role in the immune inflammation process, exhibiting various cell morphologies and functions in response to microenvironmental changes (Choi et al., 2017). To elucidate the roles of macrophages in the DRG versus the spinal cord following SCI, we investigated the subpopulations within the DRG. On the basis of our DEG findings, the macrophage subpopulations were categorized into 11 clusters, labeled MC0–10 (**Figure 3A**). The clusters constituting the majority of macrophages were MC0, MC1, and MC2. Notably, t-SNE analysis employing cluster-split-sample examination (**Figure 3B**) indicated that the following clusters were nearly absent in the spinal cord, but were significantly increased in the DRG: MC4, characterized by expression of nuclear receptor coactivator 1 (*NCOA1*) and insulin-like growth factor 1 (*IGF1*); MC6, with adiponectin, C1Q, and collagen domain containing (*ADIPOQ*), *IGF1*, and membrane associated guanylate kinase, WW, and PDZ domain containing 2 (*MAGI2*); and MC9, with polycystin 1 (*PKD1*) and *MAGI2*. The distributions of the top DEGs in MC4, MC6, MC9, and other MCs, identified as ATP binding cassette subfamily A member 6 (*ABCA6*), RNA-binding motif single-stranded interacting protein 3 (*RBMS3*), EBF transcription factor 1 (*EBF1*), laminin subunit alpha 4 (*LAMA4*), ANTXR cell adhesion molecule 2 (*ANTXR2*), laminin subunit alpha 2 (*LAMA2*), SRY-box transcription factor 5 (*SOX5*), forkhead box P2 (*FOXP2*), growth hormone receptor (*GHR*), and apolipoprotein D (*APOD*), are depicted in feature t-SNE plots (**Figure 3C**).

To assess the cellular function in the DRG following SCI, GO enrichment analysis was conducted, focusing on three categories: biological process, molecular function, and cellular component. The findings revealed the presence of small GTPase-mediated signal transduction, cytoplasmic translation, cell-matrix adhesion, regulation of cellular component size, cell-substrate adhesion, regulation of protein complex assembly, and positive regulation of transferase activity, with functions tending toward cell death regulation in the DRG (**Figure 3D**). KEGG pathway enrichment analysis of key genes in the post-SCI DRG (**Figure 3E**) highlighted pathways involving focal adhesion, cellular senescence, Rap1 signaling, FoxO signaling, and Fc gamma R-mediated phagocytosis as being the most significantly enriched.

### Subclustering of macrophages participating in the post–spinal cord injury immune response

Biological process enrichment analyses were conducted on the DEGs in each subset. Notably, clusters MC4, MC6, and MC9, which expressed a substantial number of genes with involvement in multiple functions and were found in high proportions in the DRG, emerged as multifunctional subtypes. These subtypes were found to be active in regulating axonal regeneration, leukocyte differentiation, positive regulation of gamma-delta T-cell differentiation, regulation of monocyte differentiation, and other functions. We propose that these three primary macrophage subpopulations influence the immune microenvironment of the DRG through comprehensive functional regulation.

The functions of DEGs in MC0, namely heme oxygenase 1 (*HMOX1*) and solute carrier family 1 member 3 (*SLC1A3*), were enriched in the regulation of mast cell activation, mast cell-mediated immunity, mast cell degranulation, and amino acid transport. MC1, by expressing interferon gamma receptor 1 (*IFNGR1*) and enolase 1 (*ENO1*), exhibited enrichment in the regulation of mast cell activation, mast cell-mediated immunity, mast cell degranulation, and metabolic processes. Additionally, MC8, expressing CD84, FGR proto-oncogene (*FGR*, a Src family tyrosine kinase), and *HMOX1*), showed enrichment in the regulation of mast cell activation, mast cell degranulation, regulation of leukocytes, and endothelial cell migration. The activation of afferent neurons by mast cell-derived mediators (e.g., trypsin, histamine, cysteine, leukotrienes, and neurotrophins) can result in high neuronal reactivity, thereby establishing a self-perpetuating cycle of neuroinflammation (Mukai et al., 2018). The common feature of these three subclusters is the regulation of mast cells, which may exert an inhibitory effect on the neuroinflammation microenvironment.

GO enrichment analysis indicated that subsets MC3 (Werner syndrome ATP-dependent helicase [*WRN*], MRE11 homolog, double-strand break repair nuclease [*MRE11*], RAD54 like [*RAD54L*], breast cancer 1 [*BRCA1*], and high mobility group AT-hook 2 [*HMGA2*]) and MC5 (Lyn proto-oncogene [*LYN*, an Src family tyrosine kinase], *CD74*, transforming growth factor-beta 1 [*TGFB1*], B-cell lymphoma 6 [*BCL6*], and *CD86*) may be involved in regulating the differentiation, activation, and proliferation of B cells and the cell cycle. B cells primarily function to secrete antibodies during the immune process, and can modulate macrophage polarization through the secretion of proinflammatory or anti-inflammatory factors (Tang et al., 2022). Consequently, these two subclusters may indirectly influence the immune microenvironment of macrophages via B cell mediated mechanisms.

The MC7 subset (hypoxia inducible factor 1 subunit alpha [*HIF1A*], calmodulin 1 [*CALM1*], optineurin [*OPTN*], vascular endothelial growth factor A [VEGFA], and annexin A1 [*ANXA1*]) may be involved in regulating cell death and the innate immune response. The MC10 subset (*MAGI2*, Erb-b2 receptor tyrosine kinase 4 [*ERBB4*], Erbb2 interacting protein [*ERBIN*], and GRB2 associated binding protein 1 [*GAB1*]) may participate in the ERBB signaling pathway. Laplace-Builhé et al. (2021) demonstrated that the neural crest-derived foxd3-positive cells in zebrafish regulate macrophage recruitment and polarization through the ERBB pathway, suggesting that the MC10 subcluster may influence the macrophage immune microenvironment via this pathway. The small number of enriched genes observed in MC2 indicated the lack of accumulation of relevant immune regulatory functions. Nevertheless, the key genes in MC2 (Semaphorin 5A [*SEMA5A*], integrin subunit alpha M [*ITGAM*], HIVEP zinc finger 3 [*HIVEP3*], integrin subunit alpha X [*ITGAX*], apolipoprotein E [*APOE*], DNA damage regulated autophagy modulator 2 [*DRAM2*], solute carrier family 6 member 6 [*SLC6A6*], and bromodomain adjacent to zinc finger domain 1A [*BAZ1A*]) point to its role in the immune microenvironment of macrophages. Findings by Phu et al. (2023) revealed that APOE expression in macrophages modulates the immunometabolic regulatory properties of their secreted extracellular vesicles. ITGAM and ITGAX, which are expressed in MC2, have been shown to regulate macrophage infiltration and polarization (Lou et al., 2024; Zhang et al., 2024).

### Distinct macrophage differentiation trajectory assessed through differentially expressed genes and pseudo-time analysis

**Figure 4:**
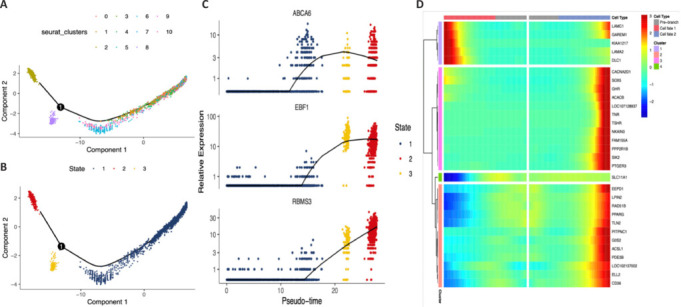
Differentiation trajectories of macrophage subclusters in the DRG following SCI in a cynomolgus monkey. (A) Differentiation trajectories of each macrophage subpopulation based on the pseudo-time analysis. (B) Differentiation trajectories of the macrophage population, color-coded by the pseudo-time process and the cell population stage. The analysis unraveled one minor bifurcation and two distinct pathways, with the cells in the right branch identified as the origin point for macrophage differentiation. (C) Differentiation trajectories of the three most highly expressed genes by macrophage state. (D) Heatmap displaying the top-ranked genes in each cluster and cell state of macrophages during the pseudo-time process. DRG: Dorsal root ganglia; SCI: spinal cord injury.

To examine the developmental insights into macrophages in the post-SCI DRG, we employed the Monocle package for pseudo-time reconstruction. In the pseudo-temporal ordering, cell subsets MC0, MC1, MC3, MC4, MC5, MC6, MC7, MC9, and MC10 are hypothesized to be among the earliest activated populations, followed by activation of MC2 (characterized by the markers *SEMA5A*, *ITGAM*, *HIVEP3*, *ITGAX*, *APOE*, *DRAM2*, *SLC6A6*, and *BAZ1A*) in state 2 (**Figure 4A**). Subsequently, MC8 (markers *CD84*, *FGR*, and *HMOX1*) exhibited a significant role in macrophage-mediated regulation of the migration of mast cells, leukocytes, and endothelial cells in state 3 (**Figure 4B**). This predicted novel state in the DRG suggests a potential shift in the macrophage microenvironment in the direction of anti-inflammation and immunosuppression. The genes *EBF1* (marker in MC6 and MC9) and *RBMS3* (marker in MC6 and MC9) were expressed at low levels at the initial stage, but increased from state 2 to the conclusion, while *ABCA6* (marker in MC6) initially exhibited low expression, but increased from state 3 onward (**Figure 4C**). *ABCA6* is postulated to be a key gene in the DRG, regulating the transition of the macrophage immune microenvironment to state 3. **Figure 4D** presents a heatmap illustrating the top-ranked genes based on clusters and macrophage cell states during the pseudo-time process.

### Cell communication analysis of cell type-dependent signaling events in the dorsal root ganglia response to spinal cord injury

**Figure 5:**
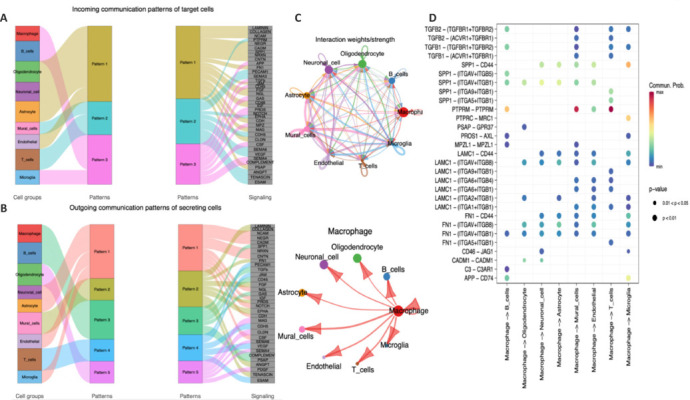
Cell communication analysis of the ligand–receptor relationship and pathways between cells in cynomolgus monkeys with SCI. (A, B) Alluvial plot of incoming (A) and outgoing (B) cell signal pattern recognition for each cell type, illustrating the intercellular communication network. (C) Circle plots showing the strength of interaction signals between different cells, and between macrophages and other cells. (D) Bubble plot showing the highly involved intercellular ligand–receptor pair pathways between macrophages and other cells. SCI: Spinal cord injury.

Cellular communication, mediated through signaling pathways, can be categorized by establishing similarity metrics and conducting multifaceted analyses from topological and functional perspectives. Our analysis identified three and five distinct patterns of incoming and outgoing signals, respectively (**Figure 5A** and **B**). These findings indicated that the majority of incoming and outgoing signals in macrophages are characterized by mode 3, comprising multiple pathways that include secreted phosphoprotein 1 (SPP1), CD45, NOTCH, TGFb, colony-stimulating factor (CSF), and gastrin (GAS). TGFb is associated with an anti-inflammatory role of macrophages, whereas activation of the NOTCH pathway results in the polarization of M1 macrophages, which exhibit a proinflammatory phenotype (Bourdon et al., 2021).

The release of microglial signaling exhibited two distinct patterns: incoming signals defined by mode 3 and outgoing signals by mode 1 (**Figure 5A** and **B**), which involves TGFb pathways, proinflammatory and macrophage CSF, and apoptosis-associated pathways (protein S (PROS1)). These findings suggested that macrophages and microglia simultaneously activate various signaling mechanisms and pathways, encompassing both pro- and anti-inflammatory responses, growth factor release, and apoptosis, among others.

CellChat analysis of nine distinct cell states pointed to macrophages as the primary communication hubs, with interaction signal intensity visualized using a circle plot (**Figure 5C**). Regarding the immune-cell group, a dot plot was used to illustrate the ligand−receptor pairs involved in intercellular communication. A bubble chart revealed the top three most active intercellular ligand–receptor pathways, demonstrating primary involvement of macrophages, B cells, microglia, T cells, neuronal cells, endothelial cells, and mural cells in immune-related pathways featuring SPP1, laminin, gamma 1 (LAMC1, also called LAMININ), and fibronectin 1 (FN1) (**Figure 5D**). SPP1, also known as osteopontin (OPN), enables neuronal cells to respond to growth factors (e.g., brain-derived neurotrophic factor [BDNF] and ciliary neurotrophic factor), facilitating a communication network across the spectrum of cell types (Liu et al., 2017). A specific set of ligand–receptor pairs were observed to be involved in the SPP1, LAMC1, and FN1 signaling pathways. Notably, the effects on microglia were predominantly mediated through three interaction pairs: SPP1-CD44, LAMC1-CD44, and FN1-CD44. These findings supported the crucial role of macrophages and microglia in the immune–inflammation process during SCI.

## Discussion

Previous research into the role of DRG neurons as donors for SCI repair has demonstrated that axons from DRG neurons exhibit robust growth and effectively promote the development of cortical neurons through a mechanism termed axon-facilitated axon regeneration (Xu et al., 2020). Furthermore, stretch-grown tissue-engineered nerve grafts, comprising stretch-grown axons spanning two populations of DRG neurons, have proven efficacious in bridging gaps in injury to peripheral nerves. SCI model rats with a full transection of 5 mm at the thoracic level were treated with DRG axons that were mechanically elongated and embedded in a collagen hydrogel, conclusively demonstrating the potential for axonal regeneration in a suitable immune microenvironment (Sadik et al., 2020). Our UMAP plots revealed a substantial increase in the proportion of neuronal cells in the DRG following SCI, while the numbers of immune-related cells, namely B cells, macrophages, microglia, oligodendrocytes, and astrocytes, decreased in the post-SCI DRG. This observation suggested that, in contrast to the aggregation of immune-related cells in the spinal cord post-SCI, the number of immune-related cells in the DRG was significantly reduced, favoring the proliferation of neuronal cells and potentially DRG axons. Additionally, our UMAP plots indicated significant increases in the proportions of endothelial and mural cells in the DRG compared with those in the spinal cord after SCI.

In the CNS, endothelial cells and mural cells form the integral components of the neurovascular unit. Vascular mural cells have been observed to contribute to scar formation following SCI (Fernández-Klett et al., 2013). After SCI, a substantial number of endothelial cells at the core of the injury site undergo apoptosis, resulting in rapid disruption of the blood–spinal cord barrier (Wang et al., 2024). Zhou et al. (2019) recently reported that microvascular endothelial cells phagocytose myelin debris through the opsonization of immunoglobulin G, a process that is dependent on the autophagy-lysosome pathway and critical for enhancing the inflammatory microenvironment during the early stages of SCI. However, Leroux et al. (2020) indicated that sensory neurons of the DRG, with the specific involvement of substance P and calcitonin gene-related peptide, remodel the extracellular matrix of endothelial cells to support and promote angiogenesis. Evidence based on a co-culture system demonstrates that human vascular endothelial cells enhance the development of DRG cells, at least in part, through the secretion of BDNF (Yuan et al., 2017).

Among non-neuronal cells, macrophages have a key role in regulating regenerative processes. Within the nerves, macrophages primarily facilitate Schwann cells in clearing debris. Under normal conditions, macrophages residing in nerves exhibit similarities to CNS microglia (Wang et al., 2020; Ydens et al., 2020). Following nerve injury, resident macrophages comprise only a small subset of cells that secrete chemoattractants to recruit macrophages derived from monocytes in the bloodstream. These recruited monocyte-derived macrophages, which express arginase 1 (ARG1), constitute the predominant macrophage population responsible for directing nerve repair (Ydens et al., 2020). However, this study found that, after SCI, the number of macrophages in the DRG dropped significantly, which subsequently facilitated neuronal cell proliferation.

The diversity of macrophages within the DRG and spinal cord tissues following SCI may influence neural recovery and the immune microenvironment. To the best of our knowledge, this is the first study to identify the heterogeneity and subsets of macrophages, and their regulatory interactions with other cells, in the post-SCI DRG of a cynomolgus monkey. Our gene enrichment analysis of macrophages categorized into 11 distinct subclusters (MC0–MC10) unraveled the 10 most highly expressed hub genes in the post-SCI DRG: *ABCA6*, *RBMS3*, *EBF1*, *LAMA4*, *ANTXR2*, *LAMA2*, *SOX5*, *FOXP2*, *GHR*, and *APOD*. In a previous study, Kaminski et al. (2001) identified upregulation of ABCA6, a new member of the ABC A-subfamily, during the differentiation of human monocytes into macrophages, suggesting a possible role for ABCA6 in macrophage lipid export. LAMA4 and LAMA2 encode components of the endothelial basement membrane laminins. The differentiation of monocytes into macrophages begins with their migration across the endothelial barrier, which consists of endothelial cells and the underlying basement membrane. Li et al. (2020) investigated the role of these laminins in the monocyte-to-macrophage transition. *FOXP2* is a member of the FOXP subfamily of transcription factors. While *FOXP1* is a crucial transcriptional regulator in the development of B cells and macrophages, *FOXP2* has been implicated in CNS development (Santos et al., 2011). Similarly, Jia et al. (2022) demonstrated that *Sox5*, a downstream target of the microRNA miR-146b-5p, plays a combined role in regulating the proinflammatory and catalytic activities of M1-like macrophages. Following nervous system injury, *APOD*, which encodes an extracellular lipid-binding protein from the Lipocalin family, is upregulated. Studies have demonstrated that axon regeneration is delayed in the absence of *APOD*, indicating its role in the early stages of Wallerian degeneration. APOD facilitates an appropriate macrophage response, contributing to the proper regulation of inflammation by ensuring that both the initiation and resolution phases of the inflammation response occur with appropriate levels and timing. This insight could contribute to the development of *APOD*-based regenerative therapies. Furthermore, Spadaro et al. (2016) provided evidence that the GHR-dependent suppression of the NOD-, LRR- and pyrin domain-containing protein 3 (NLRP3) inflammasome in macrophages contributes to pro-longevity effects by preserving immune system homeostasis during aging.

Our pseudo-temporal analysis of macrophages suggested that the MC0, MC1, MC3, MC4, MC5, MC6, MC7, MC9, and MC10 subpopulations may be the first to be activated, followed by MC2 in state 2. In subsequent state 3, MC8 was found to play a crucial role in the macrophage-mediated regulation of mast cell, leukocyte, and endothelial cell migration. These findings suggested that post-SCI DRG macrophages initially undergo activation before proceeding through a polarization program, regulated migration of mast cells, leukocytes, and endothelial cells, and ultimately transition to a phase of complement system-mediated immune regulation. Our GO enrichment results demonstrated that the indicated genes of the following subsets participate in the macrophage activation process: MC5 (complement C5a receptor 1 [*C5AR1*], C–C motif chemokine ligand 3 [*CCL3*], CD74, Fc gamma receptor IIa [*FCGR2A*], interleukin 33 [*IL33*], interleukin 4 receptor [*IL4R*], integrin subunit beta 2 [*ITGB2*], leucine rich repeat kinase 2 [*LRRK2*], protein tyrosine phosphatase receptor type C [*PTPRC*], and triggering receptor expressed on myeloid cells 2 [*TREM2*]), MC6 (CREB regulated transcription coactivator 3 [*CRTC3*], enoyl-CoA hydratase and 3-hydroxyacyl CoA dehydrogenase [*EHHADH*], forkhead box P1 [*FOXP1*], nuclear receptor subfamily 1 group H member 3 [*NR1H3*], protein kinase C epsilon [*PRKCE*], RAR related orphan receptor A [*RORA*], and strawberry notch homolog 2 [*SBNO2*]), MC7 (amyloid beta precursor protein [*APP*], hepatitis A virus cellular receptor 2 [*HAVCR2*], heat shock protein family D Hsp60 member 1 [*HSPD1*], Jun proto-oncogene [*JUN*, an AP-1 transcription factor subunit], nicotinamide phosphoribosyltransferase [*NAMPT*], and Toll-like receptor 2 [*TLR2*]), and MC9 (FOXP1, helicase, lymphoid specific [*HELLS*], low-density lipoprotein receptor [*LDLR*], leucine rich repeat and fibronectin type III domain containing 5 [*LRFN5*], mesenchyme homeobox 2 [*MEOX2*], *PRKCE*, *RORA*, *SBNO2*, and solute carrier family 7 member 2 [*SLC7A2*]). In MC2, we identified *ITGAM* (also known as *CD11b*) and *ITGAX* (also known as *CD11c*), which have indeed been demonstrated to regulate macrophage infiltration and polarization (Lou et al., 2024; Zhang et al., 2024). Lastly, our analysis revealed enrichment of *HMOX1*, which simultaneously regulates mast cell activation, leukocyte degranulation, and endothelial cell migration, in MC8. Recent studies have also emphasized the emerging significance of *HMOX1* in controlling the death of macrophage cells through ferroptosis, a process dependent on iron and oxidative stress (Yeudall et al., 2024).

Following SCI, immune cells may contribute to angiogenesis and functional recovery (Milich et al., 2021), although their interactions during angiogenesis have not been fully elucidated. Our cell communication analysis revealed that microglia and macrophages interact via the FN1, SPP1, and LAMC1 signaling pathways. *FN1*, which encodes a key member of the extracellular matrix glycoprotein family, is widely expressed by various cell types. It facilitates extracellular matrix interactions and is crucial in several developmental processes, including cell adhesion, migration, growth, and differentiation (Pankov and Yamada, 2002). Overexpression of FN1 induces the polarization of macrophages towards the M2 phenotype (Zhou et al., 2022), which may contribute to attenuation of immune inflammation in the post-SCI DRG. *LAMC1*, which encodes the laminin γ1 chain, is extensively expressed in the basement membrane and plays a role in tissue development (Aumailley, 2013). Bai et al. (2022) investigated the associations of LAMC1 expression with immune cell infiltration, as well as the expression of immunomodulatory molecules, and observed a positive correlation with the infiltration of CD4^+^ T cells, macrophages, and neutrophils. SPP1 is a phosphorylated acidic glycoprotein that regulates cell migration, adhesion, and spreading in the cardiovascular system (Pagano and Haurani, 2006). *In vitro* studies have demonstrated that SPP1 secreted by glioma cells influences endothelial progenitor cell proliferation, migration, and tube formation, thereby promoting angiogenesis (Wang et al., 2011). In our study, we found that SPP1 was secreted predominantly by macrophages for microglial regulation through CD34 receptors in the post-SCI DRG. Furthermore, our CellChat results indicated that FN1, SPP1, and LAMC1 were also secreted by macrophages to regulate endothelial cells and mural cells through CD34 receptors in the post-SCI DRG. This suggested that, in response to SCI, macrophages in the DRG likely modulate the immune microenvironment, activate angiogenesis, and promote neuronal cell proliferation by acting on endothelial cells, parietal cells, and microglia.

This study has several limitations. First, owing to the limitations of scRNA-seq, including its inability to accurately represent genes expressed at low levels, large sample sizes are necessary for reliable analysis. Second, the results of this study represent a single time point following SCI. It is well established that SCI encompasses acute, subacute, and chronic phases, necessitating sequential studies at different time points in future research. Third, the absence of studies using associated clinical specimens precludes integration with clinical data. Future research in this field should use multiomics approaches and spatial transcriptomics technology.

In summary, our scRNA-seq dataset represents the first comprehensive transcriptional analysis of the DRG following SCI in a cynomolgus monkey, encompassing the majority of cells within the DRG region. Through this dataset, we evaluated the cellular diversity within the post-SCI DRG area and investigated the signaling pathways through which macrophages associated with the immune response interact at the DRG site. Our analysis revealed novel insights into the impact of immune cells on cellular diversity and the functions of specific signaling pathways in the post-SCI DRG. These findings may help elucidate the pathophysiological mechanism underlying the response to SCI, which remains challenging to address.

**Additional file:** *Open peer review report 1.*

OPEN PEER REVIEW REPORT 1

## Data Availability

*The data are available from the corresponding author on reasonable request*.
