# Supplementary material for: Single-cell RNA sequencing of the post–spinal cord injury dorsal root ganglia in cynomolgus monkeys: Elucidation of the cellular immune microenvironment of the central nervous system
Source: Neural Regen Res. 2025 Mar 25;21(6):2506–13. doi: 10.4103/NRR.NRR-D-24-00974 (PMC13211825; doi:10.4103/NRR.NRR-D-24-00974)
Supplement: Supplementary file 1 [file NRR-21-2506_Suppl1.pdf]

OPEN PEER REVIEW REPORT 1

Name of journal: Neural Regeneration Research
Manuscript NO: NRR-D-24-00974
Title: Single-cell RNA sequencing reveals the cellular immune microenvironment of dorsal root ganglia after spinal cord injury in cynomolgus monkeys
Reviewer's Name: Palsamy Kanagaraj
Reviewer's country: USA

COMMENTS TO AUTHORS

The author investigated the cellular environment around DRG and spinal cord after SCI in cynomolgus monkeys. They have used scRNA-seq to investigate the cellular profile and to investigate macrophage heterogeneity. Immune reactions are one of the main factors that determines tissue repair after injury. Here, they have characterized for the first time the different cell types that exist in DRG and spinal cord after injury. They also have discovered different macrophage population in injured DRG. Further they have used cell trajectory and pseudo time analysis to macrophage developmental progression and ligand-receptor interaction (expression) to understand the signaling pathways they might play crucial role in immune response and inflammation.

This study provides a comprehensive data set of cellular profile and gene expression data in DRG that could be used now to analyze immune reaction and pathways that might be playing role in SCI and further process. Secondly, detailed macrophage analysis and discovered a unique sub-population (MC2 and MC8) after SCI. Also provides very specific data set for immune cell study during tissue repair to compare with regenerating animals like zebrafish. Their ligand-receptor analysis could be used to manipulate the pathways to study if the repair can be improved after SCI.

I consider this is very valuable scRNA-seq data set and analysis for further understanding in the larger animal model.
